# Effects of β-mercaptoethanol on *in vitro* maturation and glutathione level of buffalo oocytes

**DOI:** 10.14202/vetworld.2015.213-216

**Published:** 2015-02-23

**Authors:** Pankaj A. Patel, Sandhya S. Chaudhary, Gopal Puri, Virendra Kumar Singh, Arjun B. Odedara

**Affiliations:** Department of Veterinary Physiology & Biochemistry, College of Veterinary Sciences & Animal Husbandry, Navsari Agricultural University, Navsari-396450, Gujarat, India

**Keywords:** buffalo oocytes, *in vitro* maturation, β-mercaptoethanol, glutathione

## Abstract

**Aim:**

The present study was carried out to evaluate the effect of supplementation of β-mercaptoethanol (β-ME) on *in vitro* maturation rate and glutathione (GSH) level of buffalo oocytes.

**Materials and Methods:**

Oocytes were recovered from buffalo’s ovaries collected from government approved slaughter house (near Kamela darwaza, Surat) of Surat Municipal Corporation. The obtained oocytes were *in vitro* matured in maturation media supplemented with 0 μM (117 oocytes), 100 μM (46 oocytes) and 200 μM (42 oocytes) concentration of β-ME. After 24 h of incubation, maturation rate of oocytes and intra-cellular GSH level were determined.

**Results:**

The results showed that the presence of β-ME did not influence (p>0.05) the oocyte maturation rate. However, GSH level increased significantly (p<0.05) in matured oocytes when supplemented with 100 μM and 200 μM β-ME (6.19±0.10 and 6.37±0.20 pmol/oocyte) as compared to control media (4.68±0.26 pmol/oocyte).

**Conclusion:**

It was concluded that β-ME may have a potential to increase the meiotic maturation of *in vitro* cultured oocytes and protect it from oxidative damage.

## Introduction

Buffalo (*Bubalus bubalis*) plays a prominent role in rural livestock production. Problems like late onset of reproductive maturity, seasonality of breeding, late estrus and long calving interval have been attributed to poor reproductive performance of this species [1]. To cope up with these problems, use of modern biotechnologies, such as *in vitro* fertilization (IVF) and embryo production are required instead of conventional breeding programs [2].

A major factor affecting *in vitro* mammalian embryo development is increased oxidative stress [3], which is due to high lipid content of buffalo oocytes [4]. Higher amount of reactive oxygen species (ROS) can alter cellular molecules; induce developmental block, apoptosis and fragmentation of embryos [5]. It has been demonstrated that addition of low molecular thiol compound such as β-mercaptoethanol (β-ME) and cysteamine to the maturation medium causes an increase in intracellular glutathione (GSH) synthesis [6,7] and leads to low oxidative stress in many species [8].

β-ME and GSH both improve the cell survival by decreasing apoptotic cell death under “redox” state [9]. GSH directly influences cell death, while β-ME has an indirect effect by supporting increase in intracellular GSH level [5]. Buffalo oocytes can synthesize de novo during *in vitro* maturation (IVM) [7] and β-ME increases cumulus cells expansion which help in GSH synthesis [10]. So far as buffalo oocytes are concerned, meagre studies have been carried to know the effects of β-ME on cumulus expansion of oocyte and intracellular GSH content.

Therefore, the present study was carried out to evaluate the effect of supplementation of β-ME on IVM rate and GSH level of buffalo oocytes.

## Materials and Methods

### Reagents and media

All the chemical and media used in the present study were purchased from Sigma (USA).

### Collection of ovaries

Ovaries were collected from sexually matured buffaloes immediately after slaughter from nearby government approved slaughter house (near Kamela darwaza, Surat) of Surat municipal corporation and transported to the laboratory in sterile normal saline (NSS:0.85%) solution fortified with antibiotic (50 μl/L Gentamicin) at 38-39°C temperature. At laboratory, ovaries were washed in 70% ethanol for 1 min. to reduce contamination followed by washing in 0.85% NaCl twice for 1 min.

### Oocyte recovery

After final washing, cumulus oocyte complexes (COCs) were aspirated from non-atretic surface follicles (2-8 mm) using 18-guage needle connected to a 5 ml sterile syringe containing oocyte collection media. Further aspirated oocytes were searched and graded as per Khandoker *et al*. [11]. A, B and C grade oocytes were used for IVM.

### Maturation of oocytes

After final washing with oocyte collection media, oocytes of A, B and C grade were equally distributed in three groups *viz*: control Group I (117 oocytes) and treatment Groups-II (46 oocytes) and III (42 oocytes). In control group, only basic maturation media (TCM-199 supplemented with 0.2 mM sodium pyruvate, 10% fetal bovine serum, 3 mg/ml bovine serum albumin and 10 IU/ml hCG) was used while in treatment Group-II and III control media supplemented with 100 µM and 200 µM β-ME respectively was used. Before transferring to maturation media, oocytes were washed once with respective maturation media. Each group was individually placed in 50 μl droplet of maturation medium containing 5-10 oocytes covered with mineral oil in a sterile petridish and kept at 38.5°C, 5% CO_2_ and 95% humidified air in CO_2_ for 24 h.

### GSH content and maturation rate of oocytes

Maturation of oocyte assessed on the basis of their cumulus layer expansion as per Khandoker *et al*. [11]. GSH level estimation of oocytes was carried out in all groups after 24 h of maturation. Oocytes were carefully denuded by repeated pipetting, washed several times in 1x phosphate buffered saline and 10-12 oocytes from each group were stored at −20°C in eppendorf for further use. On the day of assay, the samples were thawed and 500 µl of ice-cold 5% metaphosphoric acid added to each sample. Vortexing performed for 3-5 min. After that sonication was performed for 5 min. Samples were then centrifuged (10 min; 3000 × g) in cryo-centrifuge machine at 4°C and 100 µl of supernatant was recovered. Further estimation of GSH was done with the help of GSH Assay Kit (Calbiochem^®^ USA).

### Statistical analysis

Data pertaining to oocyte maturation were analyzed by SPSS software performing Chi-square test and for data pertaining to GSH level by one-way ANOVA among control and treatment groups. A significance level of p<0.05 was used throughout this study.

## Results

The effects of supplementation of β-ME on IVM rate and GSH level of buffalo oocytes is presented in Table-1. The results showed that the presence of β-ME did not influence the oocyte maturation rate although higher maturation rate was observed in β-ME-100 and 200 μM groups as compared to control. However, intra-cellular GSH level increased significantly (p<0.05) in the presence of 100 and 200 μM β-ME (6.19±0.10 and 6.37±0.20 pmol/oocyte) as compared to control (4.68±0.26 pmol/oocyte).

**Table-1:** Effects of β-ME on of oocytes and its GSH level in buffalo.

Groups	Total no of oocytes	No. of matured oocytes (%)	GSH Level in matured oocytes (pmol/oocyte)
Control	117	78 (66.7)	4.68±0.26^a^
β-ME-100 µM	46	35 (76.1)	6.19±0.10^b^
β-ME-200 µM	42	32 (76.2)	6.37±0.20^b^

βME=βmercaptoethanol, IVM=*In vitro* maturation, GSH=Glutathione

## Discussion

Antioxidants function as autocrine and paracrine factors that influence growth, differentiation and retardation of developing follicles. Presence of GSH, β-ME is beneficial for follicle development, and there may be an interaction between exogenous antioxidant and developing follicles. Exogenous antioxidants influence follicle growth and nuclear maturation of intra-follicular oocytes. β-ME is a thiol compound, acting as an antioxidant and promotes embryo development [12,13].

The results of the present study revealed that the addition of β-ME (100 µM and 200 µM) to the maturation medium did not increase maturation rate, as also reported in porcine oocytes [14]. However, higher numbers of M-II oocytes were found when denuded oocytes were cultured in maturation medium supplemented with 25 µM β-ME [15]. Similarly, supplementation of β-ME positively influence percentage of oocytes from pre-pubertal Boer goats progressing to metaphase II stage during IVM [16]. It has also been reported that supplementation of β-ME in maturation media have positive effect on expansion of COCs and maturation rate of oocytes of bovine as well as pig [10,17], apart for impact on fertilization rate [18] and improves embryo development rate [19,20].

The effect of β-ME may have been mediated through the synthesis of GSH which is known to play an important role in protecting the cell or embryos from oxidative damage. Exogenous β-ME is able to increase GSH synthesis by reducing cystine to cysteine [21] and increased GSH level promotes embryonic development by maintaining intracellular redox state [22]. In the present study also, significantly higher GSH level was observed in groups having maturation medium supplemented with 100 µM and 200 µM β-ME than control groups. Similar results were found in bovine oocytes and embryos [7,15,22], as well as pig oocytes [12]. GSH itself plays a critical role in protecting the cell from oxidative damages [23-26]. These results warrant the supplementation of exogenous β-ME in basic oocyte maturation medium for improvement of IVF.

## Conclusion

From the present study, it can be concluded that addition of β-ME at different concentration in maturation media helps in the synthesis of GSH that protects the degeneration of oocytes from ROS during IVM and might enhance the process of maturation of oocyte.

## Authors’ Contributions

SSC along with GP designed the experiment and PAP conducted the experiment with the help of ABO. GP and VKS helped in analyzing the data and preparing the manuscript. SSC, GP, VKS and PAP reviewed the manuscript. All authors read and approved the final manuscript.
